# “It Is Comparison That Makes People Miserable”: Enterprise Social Media Visibility, Social Comparison Orientation, and Workplace Impostor Thoughts

**DOI:** 10.3390/bs16050782

**Published:** 2026-05-15

**Authors:** Chungwai So, Yixin Zhou, Juan Du

**Affiliations:** School of Business Management, Shanghai International Studies University, Shanghai 200083, China; 2038@shisu.edu.cn

**Keywords:** ESM visibility, workplace impostor thoughts, knowledge sharing, workplace well-being, social comparison orientation

## Abstract

As enterprise social media (ESM) visibility increasingly exposes employees’ work-related behaviors and competencies to organizational audiences, its potential negative psychological consequences remain underexplored. Grounded in social comparison theory and adopting the three-stage “selection, appraisal, and response” research framework, this study investigates whether and how ESM visibility fosters workplace impostor thoughts and, in turn, influences employees’ knowledge-sharing behavior and workplace well-being. Moreover, this research further examines the boundary role of social comparison orientation in shaping these effects. A two-wave, multi-source survey design was employed to test the proposed hypotheses. Data were collected from employees and their immediate supervisors in four companies across the finance, IT, management consulting, and education industries in China. To reduce common method variance, data collection was separated by a two-week interval. The final sample consisted of 447 matched employee–supervisor dyads. Hypotheses were tested using correlation and multiple regression analyses conducted in SPSS 23.0 and Mplus 8.3. Mediation and moderated mediation effects were examined using the PROCESS macro (Version 3.5) with 5000 bootstrap resamples. ESM visibility exhibited a significant positive association with workplace impostor thoughts and exerted a negative indirect effect on employees’ knowledge sharing and workplace well-being through workplace impostor thoughts. Moreover, social comparison orientation strengthens the positive effect of ESM visibility on workplace impostor thoughts, as well as the indirect effects of ESM visibility on knowledge sharing and workplace well-being via workplace impostor thoughts. The findings elucidate the relationship between enterprise social media (ESM) visibility and workplace impostor thoughts, highlighting the mediating role of workplace impostor thoughts and the moderating role of social comparison orientation. These findings suggest that ESM visibility generates unintended negative outcomes and complements research on the contextual antecedents of workplace impostor thoughts. Moreover, this study extends social comparison theory to explain employee responses to digital workplace visibility.

## 1. Introduction

In recent years, Enterprise social media (ESM), such as Alibaba’s DingTalk and Tencent’s WeCom, has garnered growing attention from both scholars and practitioners. For instance, 80% of employees in the Chinese workplace use WeChat for work-related communication among coworkers (Hong et al., 2025; Zheng et al., 2015). ESM has fundamentally transformed how employees communicate, collaborate, and share knowledge within an organization (Leonardi, 2014, 2015; Rode, 2016). According to Leonardi et al. (2013), ESMs are organizational digital platforms that enable employee communication, content sharing, and visibility in workplace interactions and relationships. Among the various affordances of ESM, visibility—defined as the extent to which employees’ activities, communications, and networks are observable to others—represents a particularly transformative feature (Treem & Leonardi, 2013). ESM visibility enables employees to access information beyond their immediate interactions, providing cues about “who knows what” and “who knows whom” within organizations (Leonardi, 2014, 2015). Therefore, ESM visibility has dramatically changed how individuals think about what it means to see others and to be seen by them (Leonardi & Treem, 2020). While prior research highlights the benefits of ESM visibility, for instance, some scholars have shown that ESM visibility can improve employee resilience and stimulate extra-role voice behavior (Deng & Zhu, 2023; Zhu et al., 2025). However, its potential psychological costs remain underexplored. Specifically, the same visibility that enhances awareness may also expose employees to constant social evaluation and comparison.

Although prior research suggests that ESM visibility helps employees rapidly develop awareness of their colleagues by inferring others’ knowledge levels from available cues, it also creates an unprecedented environment in which employees’ work behaviors, contributions, and competencies are continuously exposed to social scrutiny (Gibbs et al., 2013; Leonardi, 2014; Treem & Leonardi, 2013). Furthermore, employees can perceive “who I am” in others’ eyes and orient themselves through ESM visibility within the organization (Brzozowski et al., 2009). Exposure to others’ positive self-presentations may trigger upward social comparisons, which, in turn, foster negative self-evaluations and undermine employees’ psychological well-being (Vogel et al., 2014). Despite growing recognition of these dynamics, the potential negative psychological consequences of ESM visibility remain underexplored.

One important yet overlooked mechanism through which ESM visibility may influence employee psychology is social comparison. The abundant and easily accessible information on ESM provides fertile ground for employees to evaluate their own abilities relative to others (Vogel et al., 2014). Notably, employees tend to selectively present positive aspects of their work on ESM (Bazarova & Choi, 2014), resulting in systematically upward-biased comparison targets. Repeated exposure to colleagues’ seemingly effortless achievements and expertise may therefore trigger upward social comparisons, leading employees to perceive themselves as less competent (Guillaume et al., 2019). Such comparison processes are particularly salient for individuals high in social comparison orientation, who are more attentive to comparative information and more likely to internalize unfavorable comparisons (Buunk & Gibbons, 2005; Guillaume et al., 2019; Marder et al., 2024; Yang, 2016).

Importantly, these comparison experiences may foster workplace impostor thoughts, which are defined as persistent beliefs that one’s success is undeserved and that one will eventually be exposed as a fraud despite objective evidence of competence (Clance & Imes, 1978; Sakulku, 2011). Individuals experiencing impostor thoughts attribute their achievements to luck, timing, or interpersonal skills rather than genuine ability, and live in persistent fear of being “found out” (Clance & Imes, 1978; Vergauwe et al., 2015). While traditionally studied among high-achieving women and minority groups (Clance & Imes, 1978; Cokley et al., 2013), recent research suggests that impostor phenomenon is widespread across diverse organizational contexts and has significant implications for employee outcomes, including reduced job satisfaction, increased burnout, and impaired career development (Bravata et al., 2020; Vergauwe et al., 2015). Currently, scholars regard workplace impostor thoughts as a socially constructed and contextually triggered experience (Kark et al., 2022; Stone-Sabali et al., 2023; Tewfik et al., 2025). The academic community has redirected research on the impostor phenomenon toward the workplace to explore its impact mechanisms (Hudson & González-Gómez, 2021; Hutchins et al., 2018; Jiang et al., 2025; Tewfik, 2022; Wen et al., 2025). In environments characterized by high visibility and frequent upward social comparisons, employees may begin to question the legitimacy of their own achievements, thereby becoming more vulnerable to workplace impostor thoughts. Marder et al. (2024) found that LinkedIn users experience imposter syndrome when using the platform, and Guillaume et al. (2019) explored how social media has affected faculty sense of self through the lens of legitimacy and the impostor phenomenon. Nevertheless, whether and how organizational communication technologies, particularly ESM visibility, shape employees’ workplace impostor thoughts as a situational antecedent remains largely unexplored. Thus, the impact of ESM visibility on employees’ impostor thoughts in the workplace merits further investigation.

In addition, employees experiencing impostor thoughts may reduce knowledge sharing to maintain their organizational image, fearing exposure of their “inadequacies” (Kark et al., 2022; Sakulku, 2011). Meanwhile, continuous self-doubt and external evaluation pressure lead to psychological fatigue and emotional exhaustion, thereby diminishing employees’ well-being (Maslach & Jackson, 1981; Srivastava et al., 2006). Tewfik et al. (2025) note that although scholars have increasingly emphasized the situational and socially constructed nature of impostor thoughts in recent years, extant research continues to rely predominantly on an individual trait-based perspective. Despite these implications, prior research has rarely examined impostor thoughts as a mediating mechanism linking ESM-related experiences to employee outcomes.

To address these gaps, this study draws on social comparison theory and adopts a process perspective to examine how ESM visibility influences employee outcomes through workplace impostor thoughts. Specifically, we develop a conceptual model based on selection, appraisal, and response stages (Matthews & Kelemen, 2025). We propose that ESM visibility increases employees’ exposure to upward comparison information (selection), shapes their self-evaluations and perceptions of competence (appraisal), and ultimately triggers behavioral and emotional responses (response), including reduced knowledge sharing and lower workplace well-being. Moreover, we examine social comparison orientation as a key boundary condition that amplifies the effects of ESM visibility. ESM visibility makes employees’ communications, contributions, and accomplishments broadly observable across the organization (Treem & Leonardi, 2013), creating a rich environment for ability-based social comparisons. When employees observe colleagues’ seemingly effortless performance and expertise displayed on ESM platforms, they are likely to engage in upward social comparisons (Buunk & Gibbons, 2007), perceiving others as more competent while questioning their own abilities. This comparison process may be particularly potent in professional contexts because ESM users tend to selectively share positive work-related information (Bazarova & Choi, 2014), creating positively biased representations of colleagues’ competence that fuel feelings of relative inadequacy. Furthermore, individuals with high social comparison orientation demonstrate heightened attentional vigilance toward comparative information, greater internalization of unfavorable comparisons, and more negative affective responses following upward comparisons (Buunk & Gibbons, 2005; Vogel et al., 2014). Employees who frequently compare themselves with others and experience high self-concept uncertainty (Maslach & Jackson, 1981) are especially vulnerable to impostor thoughts (Buunk & Gibbons, 2005) when exposed to visible displays of colleagues’ accomplishments on ESM.

This study makes three main contributions. First, it advances the literature on ESM by moving beyond its predominantly positive framing and uncovering the psychological mechanism underlying how ESM visibility may generate negative outcomes. In addition, it enriches the research on ESM stressor or overload (Deng & Gan, 2026; C. Wang et al., 2023; Zhang et al., 2026). Second, it contributes to the impostor phenomenon literature by identifying ESM visibility as a novel situational antecedent, thereby shifting the focus from trait-based explanations to contextually embedded processes (Canning et al., 2020; Grenon et al., 2019; Tewfik et al., 2025). Third, by integrating social comparison theory, this study uncovers the underlying mechanism through which ESM visibility triggers workplace impostor thoughts. It extends prior research on social media and impostor thoughts, which has largely focused on general social networking contexts (Guillaume et al., 2019; Marder et al., 2024). Unlike public social media, ESM is closely tied to employees’ work performance and professional identity, thereby providing a distinct context for understanding impostor thoughts.

## 2. Literature Review and Hypothesis Development

### 2.1. Social Comparison Theory

Due to resource scarcity and fierce competition, social comparison behavior within organizations is particularly prominent. Festinger (1954) laid the cornerstone of social comparison theory (SCT), viewing social comparison as a “basic, universal, and robust psychological tendency” in humans. Gerber et al. (2018) argue that social comparison primarily focuses on two aspects: the choice of a comparison target and the effects of comparisons on self-evaluations, influence, and so forth. Accordingly, social comparison theory provides valuable insights into how individuals assess themselves by comparing various aspects of their lives to those of others. Some scholars have noted that salary comparisons, ability comparisons, and social dynamics comparisons are the three core dimensions of employee comparisons within organizations (Matthews & Kelemen, 2025). Among these, the ability dimension is the focus of this study, encompassing the knowledge and skills required for individuals to perform their role tasks, including job performance (Luffarelli et al., 2016), performance rewards (Liao et al., 2023), and overcompetence (Jahantab et al., 2023).

In social media research, while numerous studies have explored its impact on employees from a comparative social perspective, such as its influence on employee creativity (Cao et al., 2025) and job burnout (Han et al., 2020), how enterprise social media (ESM) visibility affects employees’ evaluation and perception of their own work abilities—and why it triggers workplace impostor thoughts—remains underexplored. Guillaume et al. (2019) found that social media affects faculty’s sense of self (workplace impostor thoughts), but did not explain the psychological mechanisms underlying this effect. According to the objective self-focused attention theory, Marder et al. (2024) suggest that professional SNS usage triggers impostor thoughts through heightened self-focused attention. However, professional SNS usage is typically intentional and self-directed, whereas ESM visibility is more embedded in daily work processes and involves more passive exposure to others’ activities. This distinction warrants dedicated investigation into the mechanisms through which ESM visibility shapes employees’ self-perceived competence and fosters impostor thoughts.

**Figure 1:**
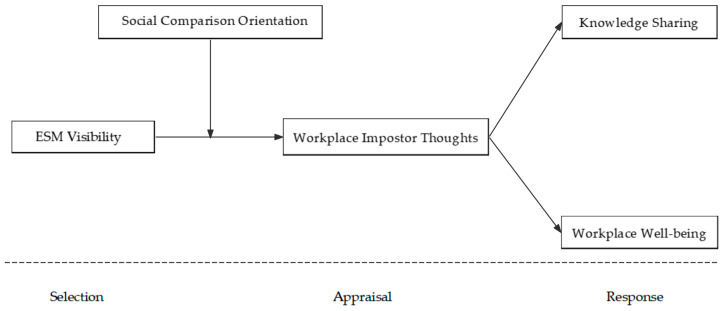
Conceptual model.

Since impostor experience emerges from social context (Gullifor et al., 2024), this study draws on an integrated model of social comparison theory proposed by Matthews and Kelemen (2025), which encompasses three stages: selection, evaluation, and response. The selection stage is jointly determined by information acquisition and comparison motivation, where information can be either actively sought or passively obtained, such as through company performance demonstrations. In the evaluation stage, the core lies in distinguishing between “reference targets” and “comparison dimensions.” Individuals often assess their own abilities and worth by comparing themselves to others, especially in situations where objective evaluation standards are lacking (Festinger, 1954; Matthews & Kelemen, 2025). In this study, as information about others’ performance, achievements, and interactions became more visible, employees were more likely to base their self-evaluation on relative rather than absolute standards. This highly visible information environment lowered employees’ self-perception of their abilities, and this gap translated into impostor thoughts when they recognized that others still held them in high regard. During the response phase, the final psychological and behavioral outcomes manifested as assimilation or contrast responses—the former involving movement toward the comparison target and the latter involving deliberate distancing. This theoretical framework guides the present study in understanding self-evaluation and the formation of employees’ subsequent psychological and behavioral patterns. The theoretical model is depicted in Figure 1.

### 2.2. ESM Visibility and Workplace Impostor Thoughts

Building on the selection stage of Matthews and Kelemen’s (2025) integrated model, which involves information acquisition and comparison motivation, prior research has demonstrated that social comparison is pervasive across various domains and is particularly salient in organizational contexts, where employees frequently evaluate their standing relative to their coworkers (Matthews & Kelemen, 2025). In traditional work environments, social comparisons revolve around in-person interactions with close others, such as coworkers and friends (Vogel et al., 2014). However, virtual environments not only expand the scope of social comparison to include employees from different work teams or departments (Greenberg et al., 2007) but also amplify comparative tendencies owing to the easy and ubiquitous availability of information (Gomez et al., 2022; Vogel et al., 2014). Within this framework, individuals construct self-evaluations through a two-stage process involving the selection of comparison targets and the appraisal of their own abilities. ESM visibility increases employees’ exposure to coworkers’ work-related interactions and achievements, thereby providing fertile ground for social comparison.

ESM visibility enables employees to observe not only coworkers’ work-related communications but also their social connections and interaction patterns (Leonardi, 2014, 2015; Oostervink et al., 2016; Treem et al., 2020). In organizational contexts, ESM visibility represents a dual-edged phenomenon. On the one hand, it enhances others’ perceptions of an employee’s competence and allows observers to infer coworkers’ expertise and capability levels (Chen & Wei, 2019; Leonardi, 2014; Treem & Leonardi, 2013). On the other hand, ESM visibility may amplify concerns about competence inadequacy and the exposure of personal shortcomings, thereby undermining collaboration among employees (Arazy & Gellatly, 2012; Kane, 2017). More fundamentally, ESM visibility creates a highly exposed online social environment in which employees are encouraged to engage in intensified self-presentation, continuously monitor their own behaviors, and endure persistent evaluation and scrutiny conditions that are particularly conducive to the emergence of workplace impostor thoughts. Tewfik (2022) defines workplace impostor thoughts as the belief that others overestimate one’s competence at work, representing a discrepancy between how one thinks others perceive them and how one perceives oneself (Gullifor et al., 2024).

The appraisal stage of the social comparison model concerns both the comparison target and the dimensions of comparison (Matthews & Kelemen, 2025). Specifically, it matters who the actor compares with and what they compare on (Matthews & Kelemen, 2025). Research suggests that individuals rely on social referents to assess their abilities and to understand their relative standing within the organization (Buunk & Gibbons, 2007; Bazarova & Choi, 2014; Buunk & Gibbons, 2005; Festinger, 1954; Helgeson & Mickelson, 1995; Matthews & Kelemen, 2025). Through ESM visibility, employees gain detailed insight into coworkers’ professional competencies, personal interests, and social interactions (Chen & Wei, 2019; Leonardi, 2014, 2015), which facilitates continuous comparisons regarding whether one is “more capable” than others. Meanwhile, employees are highly attentive to how they are perceived by others and are particularly sensitive to external evaluations (Bolino et al., 2016; Leary et al., 2000). As an internally transparent and persistent communication platform, ESM intensifies these evaluative concerns. Heightened visibility can deepen employees’ self-doubt, fostering the belief that others may be overestimating their competence and increasing fears of exposing personal weaknesses or deficiencies (Arazy & Gellatly, 2012; Hudson & González-Gómez, 2021; Kark et al., 2022). Deng and Gan (2026) further argue that increased visibility may undermine employees’ self-confidence. Importantly, these comparison-induced reductions in self-evaluation do not necessarily align with stable external evaluations within the organization. As a result, employees may simultaneously perceive that others regard them as competent while they themselves feel less capable, thereby generating a discrepancy between self-perception and perceived external evaluation. According to Gullifor et al. (2024), such self–other evaluative misalignment constitutes the core cognitive basis of impostor thoughts. Consequently, ESM visibility is likely to exacerbate employees’ workplace impostor thoughts. Thus, we hypothesize the following:

**H1.**  *ESM visibility is positively related to workplace impostor thoughts.*

### 2.3. Implications for Knowledge Sharing and Workplace Well-Being

The response phase of the social comparison model encompasses various actor responses (Matthews & Kelemen, 2025; Mussweiler & Strack, 2000). Individuals do not merely engage in cognitive evaluations after social comparison; they also exhibit corresponding psychological and behavioral reactions. Knowledge sharing can be defined as a social interaction culture involving the exchange of employee knowledge, experiences, and skills throughout the department or organization (Lin, 2007). Prior research has indicated that leaders experiencing impostor thoughts may maintain distance from their subordinates and avoid sharing complete information or knowledge with employees (de Vries, 2005; L. Wang et al., 2024). While ESM visibility facilitates informal collaboration within organizations, supporting knowledge sharing and mitigating knowledge sabotage (Kosonen & Kianto, 2009; J. Wang et al., 2025), the information overload it generates can simultaneously distract employees’ attention and impede communication and mutual learning (Gibbs et al., 2013).

Employees assign labels such as “expert” or “highly competent” to others based on the positive content and comments posted on ESM (Leonardi & Treem, 2012). Those employees who are labeled as such are prone to engage in social comparison during online interactions with colleagues and simultaneously worry about exposing their own inadequacies, thereby developing workplace impostor thoughts (Ang et al., 2003; Arazy & Gellatly, 2012). Fearing that leaders and colleagues will discover their “fraudulence” and perceive them as lacking competence (Sakulku, 2011), these individuals subsequently reduce their knowledge-sharing behaviors in an effort to maintain a favorable personal image.

**H2.**  *ESM visibility has a negative indirect effect on knowledge sharing via workplace impostor thoughts.*

According to the response phase of social comparison theory (Matthews & Kelemen, 2025), workplace well-being encompasses work satisfaction and work-related affect (Zheng et al., 2015), providing a comprehensive reflection of employees’ psychological states at work. Research indicates that individuals experiencing impostor phenomenon tend to exhibit pessimistic attitudes toward their future work performance (Baumeister & Jones, 1978; Schlenker, 1975), and employees who have experienced impostor syndrome demonstrate diminished well-being (Srivastava et al., 2006; Swaidan & Jabbour Al Maalouf, 2025; Vergauwe et al., 2015). A meta-analysis further revealed that impostor phenomenon predicts lower job satisfaction, increased burnout, and reduced overall well-being (Bravata et al., 2020). Drawing on social comparison theory (Matthews & Kelemen, 2025), ESM visibility enables employees to observe their colleagues’ communication activities and work behaviors (Leonardi, 2014, 2015), creating a rich information environment for social comparison. When employees observe their colleagues’ seemingly effortless performance through ESM, they are likely to engage in upward social comparison—perceiving others as more competent while questioning their own abilities (Buunk & Gibbons, 2007). This dynamic is further exacerbated by selective self-presentation on ESM, which may create inflated perceptions of others’ competence (Chou & Edge, 2012). Consequently, individuals may struggle to derive a sense of value from their work, which in turn erodes their workplace well-being and diminishes both work satisfaction and overall life satisfaction.

**H3.**  *ESM visibility has a negative indirect effect on workplace well-being via workplace impostor thoughts.*

### 2.4. The Moderating Effect of Social Comparison Orientation on the Relationship Between Knowledge Sharing and Workplace Well-Being

Drawing on social comparison theory (Matthews & Kelemen, 2025), the primary goal of social comparison is to acquire information about the self. As one of the fundamental motivations underlying social comparison, self-evaluation primarily focuses on opinions and abilities (Gibbons & Buunk, 1999). Specifically, individuals high in social comparison orientation (SCO) demonstrate particular sensitivity to social comparison information, and this sensitivity significantly influences their self-evaluation, self-esteem, and overall psychological well-being (Buunk & Gibbons, 2005; Gibbons & Buunk, 1999). Those high in SCO seek out more comparisons, spend more time engaging in comparisons, and experience stronger emotional reactions from comparing themselves with others (Buunk & Dijkstra, 2014). In the social media context, SCO is particularly important because SNSs provide rich opportunities for social comparison, which can influence one’s psychological well-being (Yang, 2016). For example, high-SCO individuals may exhibit worse self-perception, lower self-esteem, and stronger negative emotions after browsing social media platforms, precisely because they are more attuned to the abundant comparative information available (Vogel et al., 2015).

ESM visibility makes employees’ online discussions, contributions, and publicly posted information accessible to virtually all organizational members, thereby providing a rich stream of comparative information about colleagues’ competencies, expertise, and achievements (Leonardi, 2014; Oostervink et al., 2016; Treem & Leonardi, 2013). Research demonstrates that high-SCO individuals experience more negative affective responses and lower self-esteem following exposure to upward comparison information (Vogel et al., 2014), which aligns with the core phenomenology of impostor syndrome—persistent self-doubt despite objective evidence of competence (Clance & Imes, 1978). When ESM renders work processes and outcomes highly visible, particularly when high-SCO individuals are exposed to colleagues who appear more accomplished or competent, they are more prone to internalize these unfavorable comparisons. Moreover, such individuals are reluctant to receive negative evaluations; they fear exposing their shortcomings and worry that others will perceive them as inadequate (Clance & Imes, 1978; Gardner et al., 2019). In contrast, such cognitive distortions are less prevalent among low-SCO individuals because they demonstrate lower sensitivity to social evaluative information and are better able to rationally assess how they are perceived by others (Buunk & Gibbons, 2005). In sum, while heightened ESM visibility increases employees’ exposure to others’ achievements and thereby triggers social comparison processes, the strength of this effect is moderated by individual SCO levels. Employees with high SCO are more likely to internalize comparison information, leading to stronger workplace impostor thoughts, whereas employees with low SCO are less affected owing to their lower propensity to engage in social comparisons. On this basis, we hypothesize the following:

**H4.**  *Social comparison orientation will moderate the relationship between ESM visibility and workplace impostor thoughts, such that this positive relationship will be stronger under conditions of a highly social comparison orientation.*

### 2.5. Conditional Indirect Effects

Given the moderation hypotheses and the premise that ESM visibility shapes employees’ workplace impostor thoughts, social comparison orientation is also likely to affect the strength of the indirect relationship between ESM visibility and knowledge sharing as well as workplace well-being. Based on Hypotheses 2–4 and drawing on social comparison theory, we further propose that social comparison orientation moderates the indirect effects of ESM visibility on knowledge sharing and workplace well-being via workplace impostor thoughts may be moderated by social comparison orientation. Specifically, when the level of social comparison orientation is higher, ESM visibility will intensify workplace impostor thoughts, thereby leading to reduced knowledge-sharing behaviors and diminished workplace well-being. Therefore, we propose the following conditional indirect effects hypotheses:

**H5.**  *The indirect effect of ESM visibility on knowledge sharing via workplace impostor thoughts will be moderated by social comparison orientation, such that this negative indirect effect will be stronger under conditions of a highly social comparison orientation.*

**H6.**  *The indirect effect of ESM visibility on workplace well-being via workplace impostor thoughts will be moderated by social comparison orientation, such that this negative indirect effect will be stronger under conditions of a highly social comparison orientation.*

## 3. Materials and Methods

### 3.1. Participants

Given that domestic enterprises and organizations commonly use enterprise social media (such as WeCom (Version 4.1.39) and DingTalk (Version 7.8.10)) as a medium and tool for employee communication, this study chose to conduct data collection in China. In the initial stage of the survey, we contacted six companies, and four agreed to participate. The two companies in Shanghai were a listed investment holding company and a well-known headhunting firm; the two companies in Xi’an were an educational publishing company and a Singaporean foreign-invested enterprise in the High-tech Zone. All four companies are medium-sized enterprises. Then we provided HR with an overview of the research and clarified the research objectives. The HR of four of these companies explicitly stated that employees were required to perform all work communication, information sharing, and task implementation tasks using ESM platforms. However, the Xi’an-based IT company had its own ESM platform. In addition, there was no particular department that preferred ESM. Employees in four companies were required to work in the office. Company regulations explicitly mandated daily clock-in, and remote work was only supported in special circumstances such as pandemics.

Before the survey, we contacted HRs at four companies in Shanghai and Xi’an to help distribute the online survey within their companies. We sent the online survey link to each company’s HR department, which then forwarded it to the employees. Participants were selected from different departments, such as promotion, strategic investment, and operations. Referring to the study by Wen et al. (2025), we collected data via a two-wave survey, with each interval lasting two weeks to reduce the potential impact of common method variance (Podsakoff et al., 2003; Podsakoff et al., 2012). At Time 1, 500 participants were invited to provide ESM visibility, social comparison orientation, and demographic information. A total of 482 participants responded, obtaining a response rate of 96.40%. Two weeks later (Time 2), we invited these 482 participants to report on workplace impostor thoughts and workplace well-being. With the assistance of the HR managers of the four companies, we contacted the immediate supervisors of the respondents and asked them to rate each of their employees on knowledge sharing and send their responses directly to our emails. A final total of 447 participants responded, achieving a response rate of 92.74%.

Among the participants, 158 (35.35%) were male, and 289 (64.65%) were female. The majority of participants were aged between 31 and 35 years (29.98%). Regarding education, 296 participants (66.22%) held bachelor’s degrees. In terms of total work experience, 127 participants (28.41%) had 6–10 years of professional experience. Regarding organizational tenure, 135 participants (30.20%) had been employed at their current organization for 3–5 years. In total, 298 participants (66.67%) were non-management employees. Finally, on average, 123 participants (27.52%) spent approximately 2–3 h daily using ESM for work-related interaction. Detailed demographic characteristics are presented in Table 1.

### 3.2. Measures

Before conducting the survey, we followed the standard procedure to ensure the accuracy of the questionnaire when translated from English to Chinese (Brislin, 1980). In addition, a panel of experts was then invited to review the survey items and provide feedback and suggestions, which were used to make certain clarity and readability. All items were measured using a seven-point Likert scale, ranging from 1 (strongly disagree) to 7 (strongly agree).

#### 3.2.1. ESM Visibility

The ESM visibility scale developed by Rice et al. (2017) was adopted. This scale is composed of three items, including “Enterprise social media (e.g., WeCom, DingTalk) enables me to see who has interactions or links with particular employees or their information”. The Cronbach’s *α* in this study was 0.771.

#### 3.2.2. Social Comparison Orientation

Social comparison orientation was measured using a six-item scale adapted from the Iowa–Netherlands Comparison Orientation Measure (INCOM) (Gibbons & Buunk, 1999). Participants responded to six items (e.g., “I often compare myself with others with respect to what I have accomplished.”) on a 7-point Likert scale. The Cronbach’s α in this study was 0.756.

#### 3.2.3. Workplace Impostor Thought

The scale adapted by Tewfik (2022) for measuring workplace impostor thoughts was adopted. The scale consists of five items, including “At work, people important to me think I am more capable than I think I am.” The Cronbach’s *α* in this study was 0.899.

#### 3.2.4. Knowledge Sharing

Subordinates’ knowledge sharing was assessed by supervisors using the adapted eight-item scale of Bartol et al. (2009). A sample items is “This employee readily passes along information that may be helpful to the work “. The Cronbach’s *α* in this study was 0.932.

#### 3.2.5. Workplace Well-Being

The workplace well-being scale developed by Zheng et al. (2015) was adopted. It was assessed via six items. Sample items include “I feel basically satisfied with my work achievements in my current job” and “I am satisfied with my work responsibilities.” The Cronbach’s *α* in this study was 0.874.

#### 3.2.6. Control Variables

The control variables included gender, age, educational level, job position, total work experience, organizational tenure, and ESM use hours. Prior research suggests that these variables may be associated with knowledge sharing and workplace well-being (J. Wang et al., 2017).

## 4. Results

### 4.1. Common Method Bias

Firstly, a time-lagged research design with multiple data collection points was adopted to reduce the potential influence of common method bias (Podsakoff et al., 2003). Secondly, to assess the potential influence of common method bias (CMB) in this study, Harman’s single-factor test was performed following established procedures (Podsakoff et al., 2012; Richardson et al., 2009). Results from the unrotated exploratory factor analysis revealed that no single dominant factor emerged, as the largest factor explained only 30.651% of the total variance, which is below the commonly accepted 40% threshold. Thirdly, to examine the discriminant validity of the study variables, confirmatory factor analysis (CFA) was conducted in Mplus 8.3. Results indicated that the hypothesized five-factor model provided a good fit to the data (χ^2^ = 706.52, df = 340, χ^2^/df = 2.078, CFI = 0.980, TLI = 0.976, RMSEA = 0.049, SRMR = 0.026). Moreover, this model demonstrated a significantly better fit than all alternative models, providing support for the distinctiveness of the focal constructs. Table 2 presents the CFA results for the measurement models.

### 4.2. Preliminary Analysis

Table 3 presents the means, standard deviations, and correlations among the primary study variables. As shown in Table 3, ESM visibility was positively related to workplace impostor thoughts (*r* = 0.154, *p* < 0.01), providing preliminary support for Hypothesis 1. Furthermore, workplace impostor thoughts were negatively associated with employee knowledge sharing (*r* = −0.330, *p* < 0.01) and workplace well-being (*r* = −0.275, *p* < 0.01). Thus, these findings provide preliminary support for the study’s hypotheses.

### 4.3. Mediation Analysis

We conducted path analysis using Mplus 8.3. to examine the hypotheses and utilized the PROCESS macro (Version 3.5) to conduct 5000 bootstrap tests for assessing mediation effects. Table 4 presents path analysis results for main effects and mediation effects. After considering all variables, ESM visibility was positively related to workplace impostor thoughts (*b* = 0.264, *p* < 0.001); thus, Hypothesis 1 was supported. To further examine the proposed mediation effects, a bootstrap analysis using 5000 resamples was conducted (Jj & Af, 2021). ESM visibility had a significant indirect effect on knowledge sharing through workplace impostor thoughts (estimate = −0.0520, 95% CI [−0.0895, −0.0182]). Meanwhile, ESM visibility had a significant indirect effect on workplace well-being through workplace impostor thoughts (estimate = −0.0429, 95% CI [−0.0773, −0.0419]). Thus, Hypothesis 2 and Hypothesis 3 were supported.

### 4.4. Moderated Mediation Analysis

**Figure 2:**
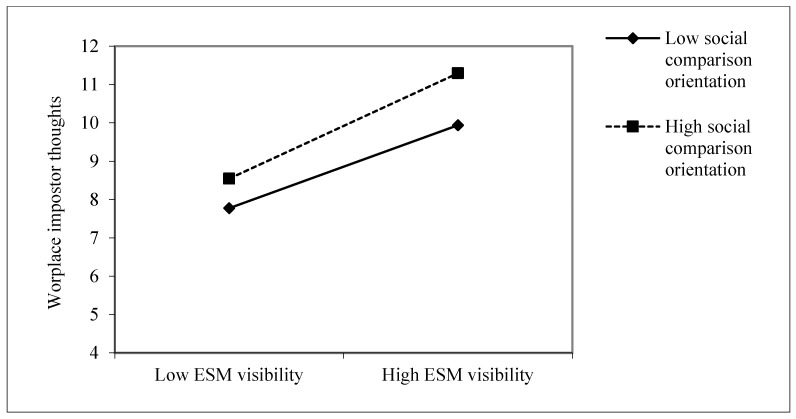
The interaction effect of ESM visibility and social comparison orientation on workplace impostor thoughts.

With respect to the moderating effect, as shown in Table 5, the interaction between ESM visibility and social comparison orientation had a positive effect on workplace impostor thoughts (*b* = 0.123, *p* < 0.01), demonstrating that social comparison orientation significantly moderated the relationship between ESM visibility and workplace impostor thoughts. Additionally, simple slopes analysis (see Figure 2) revealed that the effect of ESM visibility on workplace impostor thoughts was more positive at higher than lower levels of social comparison orientation. Therefore, Hypothesis 4 is supported.

As shown in Table 6, for knowledge sharing, the results of bias-corrected bootstrapping demonstrated that the indirect of ESM visibility on knowledge sharing through workplace impostor thoughts was more negative at higher (+1SD; estimate = −0.106, 95% CI [−0.159, −0.056]) than lower (−1SD; estimate = −0.012, 95% CI [−0.054, 0.029]) levels of social comparison orientation. Meanwhile, for workplace well-being, the results of bias-corrected bootstrapping demonstrated that the indirect of ESM visibility on workplace well-being through workplace impostor thoughts was more negative at higher (+1SD; estimate = −0.087, 95% CI [−0.137, −0.044]) than lower (−1SD; estimate = −0.010, 95% CI [−0.048, 0.022]) levels of social comparison orientation. Thus, Hypothesis 5 and Hypothesis 6 were supported.

## 5. Discussion

The research findings contribute to three important insights. First, based on the social comparison theory, this study explores the positive correlation between ESM visibility and workplace impostor thoughts. Scholars have noted that the situational origins of the impostor phenomenon have been largely neglected (Ellison et al., 2015; Kark et al., 2022). This study responds to scholarly calls for research on the impostor phenomenon and extends existing research on its antecedents in the ESM context. Meanwhile, this study advances our understanding of how digital communication environments shape employees’ self-perceptions and psychological experiences. Second, this study offers a perspective by demonstrating the impact of ESM visibility on knowledge sharing and workplace well-being through workplace impostor thoughts. Previous scholars have predominantly emphasized that ESM facilitates knowledge sharing among employees (Leonardi & Treem, 2012; Rode, 2016). Our study reveals that employees experiencing the impostor phenomenon reduce knowledge-sharing behaviors due to concerns about exposing their perceived shortcomings. Last but not least, drawing on the social comparison theory (Matthews & Kelemen, 2025), the study examines how social comparison orientation positively moderates the relationship between ESM visibility and workplace impostor thoughts, as well as the indirect effect of ESM visibility on knowledge sharing and workplace well-being.

### 5.1. Theoretical Contributions

First, extant research has examined the adverse effects of digital stress, ESM use, and ESM overload through an affective perspective. For instance, Ma et al. (2024) demonstrated that work-related social media usage exerts a detrimental influence on employee emotional exhaustion. Building on this affective perspective, Li and Liu (2025) further argued that ESM usage may amplify workplace fear of missing out (WFoMO), defined as the anxiety arising from the perception that one may miss rewarding work-related experiences or opportunities (Budnick et al., 2020), which in turn shapes employees’ attitudes and behaviors. Distinct from this affective stream of research, this study adopts a cognitive perspective to investigate how ESM usage undermines employees’ perceptions of their own success at work. Specifically, we draw on the concept of workplace impostor thoughts, defined as employees’ beliefs that others overestimate their competence (Tewfik, 2022), as a cognitive pathway through which ESM usage produces harmful outcomes. By shifting the analytical focus from affective states to cognitive self-perceptions, this study extends the ESM literature and offers a more complete account of how digital work tools shape employee psychology.

Second, this study advances social comparison theory by relocating it into digitally visible organizational contexts. Although recent work has called for greater attention to social comparison in hybrid work and virtual environments (Cao et al., 2025; Matthews & Kelemen, 2025; Whelan et al., 2026), research has yet to explain how such processes unfold in ESM settings. Drawing on the selection–appraisal–response framework, we theorize that ESM visibility transforms social comparison into an ongoing evaluative process by making cues about coworkers’ expertise, interactions, and persistently observable standing available. Employees thus form judgments about their own abilities based on visible signals of “who knows what” and “who knows whom.” By showing how digital visibility infrastructures shape self-evaluation, this study extends social comparison theory and provides a new perspective on the psychological consequences of ESM. In addition, prior research suggests that virtual environments deny employees some comparative information that might otherwise be available in traditional work environments (Greenberg et al., 2007). This study introduces social comparison orientation as a key boundary condition, deepening our understanding of social comparison theory. We demonstrate that ESM communication visibility reconstructs the social comparison context by making coworkers’ interactions, achievements, and expertise more observable (Leonardi, 2014, 2015; Treem & Leonardi, 2013).

Third, this study contributes to the impostor phenomenon literature by integrating social comparison theory with workplace impostor thoughts and uncovering a novel context-specific formation mechanism in digital work environments. Whereas prior research has largely explained impostor thoughts in terms of personality traits and early life experiences (Clance & Imes, 1978; Grenon et al., 2019; Vergauwe et al., 2015), this study demonstrates that ESM visibility constitutes an important situational trigger in contemporary workplaces. By showing that workplace impostor thoughts mediate the relationship between ESM visibility and key employee outcomes, this study extends impostor phenomenon research from individual predispositions to technology-enabled organizational contexts. In addition, it identifies a distinct psychological pathway through which impostor thoughts reduce knowledge-sharing behavior and undermine workplace well-being, thereby enriching both impostor phenomenon theory and digital workplace research. This finding resonates with but extends beyond Rode’s (2016) observation that low self-efficacy inhibits knowledge contribution.

### 5.2. Practical Implications

Based on the above findings, this study offers the following implications for organizational management practice. First, the findings demonstrate that excessive ESM use triggers workplace impostor thoughts and undermines both knowledge sharing and job well-being. This suggests that the prevailing organizational assumption that “more communication is better” may be misguided in digital contexts (Yu et al., 2018). Managers need to recognize that ESM is not a panacea and that over-reliance on digital communication tools can paradoxically harm employee psychological health and productive behaviors. By establishing and exemplifying healthy ESM usage patterns, organizations can help employees maintain work–life boundaries and reduce the psychological burden associated with persistent digital presence.

Second, organizations must proactively address impression management pressures inherent in digital communication environments. Our findings reveal that ESM visibility amplifies employees’ concerns about how they are perceived by others, contributing to impostor thoughts and psychological distress. Organizations should cultivate psychologically safe communication climates that encourage authenticity and vulnerability in digital interactions (Edmondson, 1999). Leaders can model psychological safety by openly sharing their own challenges, uncertainties, and learning experiences through ESM platforms, thereby normalizing imperfection and reducing the pressure employees feel to project flawless professional personas (Detert & Edmondson, 2011). In addition, this suggests that organizations should adopt a nuanced approach to visibility rather than automatically opting for maximum transparency (Leonardi, 2014). For communications involving sensitive information, personal development, or exploratory ideas, organizations should provide private or semi-private channels that reduce evaluative pressure and foster psychological safety (Treem & Leonardi, 2013).

Third, our findings highlight the importance of accounting for individual differences in social comparison orientation when implementing and managing ESM platforms. Employees with a high social comparison orientation are more likely to attend to and be influenced by visible comparison cues embedded in ESM interactions, which may increase feelings of self-doubt and impostor thoughts. Managers can mitigate these risks by providing guidance and training that help employees interpret coworkers’ visible achievements as sources of learning and inspiration rather than as strict performance benchmarks. Such interventions may be especially beneficial for individuals who are prone to frequent social comparison (Gibbons & Buunk, 1999).

### 5.3. Limitations and Future Directions

Despite making certain theoretical and practical contributions, this study has the following limitations that need to be addressed in future research. First, this study does not differentiate between work-related and social-related ESM usage, nor does it distinguish between public social media (PSM) and enterprise social media (ESM). This represents a significant limitation, as emerging research suggests that different types of ESM use exert distinct effects on employees (Li & Liu, 2025). For example, work-related ESM use may therefore interrupt employees’ daily lives, while social-related ESM use initially increases information overload (Chen & Wei, 2019). Indeed, these distinct usage patterns may trigger different psychological mechanisms and produce varying effects on workplace impostor thoughts and employee outcomes. Future research should explicitly distinguish among these platform types and usage purposes, examining how work-related versus social-related usage and ESM versus PSM engagement differentially influence workplace impostor thoughts.

Second, future research should incorporate multiple data sources and measurement approaches to enhance construct validity and reduce method bias. Additionally, experience sampling methods would enable researchers to capture within-person fluctuations in ESM usage, impostor thoughts, and outcomes over time, thereby illuminating dynamic processes more definitively than cross-sectional designs (Beal, 2015; Ohly et al., 2010).

Finally, the sample of this study primarily comprises employees from organizations in China, raising questions about the cross-cultural generalizability of the findings. Cultural values and norms significantly influence individuals’ orientation towards technology use, impression management motivations, and psychological responses to workplace challenges (Hofstede, 2001; Taras et al., 2010). For instance, collectivist cultures, which are characteristic of many Asian societies, including China, emphasize group harmony, social interdependence, and sensitivity to others’ evaluations (Markus & Kitayama, 1991). Consequently, the relationships between ESM visibility, workplace impostor thoughts, and outcomes may be more pronounced in collectivist contexts than in individualistic Western cultures, where self-reliance and individual achievement are more highly regarded (Cokley et al., 2013). Future research should conduct cross-cultural comparisons to test the boundary conditions of this study’s theoretical model across diverse cultural contexts.

## Figures and Tables

**Table 1:** Sample characteristics (n = 447).

Variables	Frequency	Percentage
**Gender**		
Male	158	35.35%
Female	289	64.65%
**Age**		
≤25 years	63	14.09%
26–30 years	112	25.06%
31–35 years	134	29.98%
36–40 years	93	20.81%
≥41 years	45	10.06%
**Education**		
High school or below	47	10.51%
Bachelor’s	296	66.22%
Master’s	89	19.91%
Doctorate	15	3.36%
**Work Experience**		
<1 year	32	7.16%
1–2 years	56	12.53%
3–5 years	112	25.06%
6–10 years	127	28.41%
>10 years	120	26.84%
**Organizational tenure**		
<1 year	39	8.72%
1–2 years	93	20.81%
3–5 years	135	30.20%
6–10 years	128	28.64%
>10 years	52	11.63%
**Position**		
Non-management employee	298	66.67%
Manager	78	17.45%
Senior or executive manager	35	7.83%
Others	36	8.05%
**ESM usage (hours per day)**		
<0.5 h	14	3.13%
0.5–1 h	26	5.82%
1–2 h	104	23.27%
2–3 h	123	27.52%
3–4 h	84	18.79%
>4 h	96	21.47%

**Table 2:** Model fits of measurement models (n = 447).

Models	χ^2^	df	χ^2^/df	CFI	TLI	RMSEA	SRMR
Five-factor model ^A^	706.52	340	2.078	0.980	0.976	0.049	0.026
Four-factor model ^B^	1032.84	344	2.999	0.955	0.949	0.067	0.041
Three-factor model ^C^	1456.73	347	4.198	0.918	0.908	0.085	0.061
Two-factor model ^D^	2162.91	349	6.197	0.865	0.849	0.108	0.087
Single-factor model ^E^	3324.37	350	9.498	0.776	0.748	0.138	0.123

^A^: In this model, all items were influenced by their own factors, respectively. ^B^: In this model, items for knowledge sharing and workplace well-being were influenced by the same factor, and items for other variables were influenced by their own factors, respectively. ^C^: In this model, items for knowledge sharing and workplace well-being were influenced by the same factor; ESM visibility and social comparison orientation were influenced by the same factor. ^D^: In this model, items for knowledge sharing and workplace well-being were influenced by the same factor; ESM visibility, social comparison orientation, and workplace impostor thoughts were influenced by the same factor. ^E^: In this model, there is only one factor influencing all variables.

**Table 3:** Descriptive statistics and correlations (n = 447).

Variables	Mean	SD	1	2	3	4	5	6	7	8	9	10	11	12
1. Gender	1.65	0.479												
2. Age	2.72	1.133	−0.124 **											
3. Education	2.05	0.510	0.070	0.005										
4. Work Experience	3.60	1.154	−0.115 **	0.854 **	−0.031									
5. Organizational tenure	3.17	1.091	−0.116 **	0.758 **	−0.013	0.850 **								
6. Position	1.90	0.990	0.087	0.530 **	0.198 **	0.497 **	0.469 **							
7. ESM usage (hours per day)	4.19	1.329	0.049	0.168 **	0.062	0.152 **	0.119 **	0.207 *						
8. ESM Visibility	4.790	1.019	−0.043	0.056	0.011	0.061	0.090 *	−0.007	0.462 **	(0.771)				
9. Social Comparison Orientation	5.596	0.896	−0.132 **	0.050	0.007	0.100 *	0.101 *	0.049	0.155 **	0.088	(0.756)			
10. Workplace Impostor Thoughts	3.472	1.110	0.049	−0.115 *	−0.007	−0.167 **	−0.179 **	−0.058	−0.077	0.154 **	−0.166 **	(0.899)		
11. Knowledge Sharing	5.605	0.100	−0.086	0.169 **	0.035	0.222 **	0.273 **	0.128 *	0.076	0.004	0.320 **	−0.330 **	(0.932)	
12. Workplace Well-being	5.708	0.855	−0.079	0.209 **	0.020	0236 **	0.249 **	0.204 **	0.043	−0.014	0.463 **	−0.275 **	0.605 **	(0.874)

Notes: * *p* < 0.05 (two-tailed), ** *p* < 0.01 (two-tailed). Reliability is provided on the diagonal. Abbreviation: SD, standard deviation.

**Table 4:** Path analysis results for main effects and mediation effects.

Variables	Workplace Impostor Thoughts (T2)		Knowledge Sharing (T2)		Workplace Well-Being (T2)	
	*b*	*SE*	*b*	*SE*	*b*	*SE*
**Control Variables**						
Gender	0.091	0.044	−0.122	0.089	−0.127	0.091
Age	0.114	0.129	−0.122	0.089	−0.127	0.091
Education	−0.044	−0.022	−0.092	0.073	−0.014	0.074
Experience	−0.108	−0.125	0.066	0.084	0.000	0.085
Organizational Tenure	−0.184 *	−0.201	0.011	0.086	0.036	0.088
Position	0.073	0.073	0.243 **	0.073	0.103	0.075
ESM Use Hours	−0.146 ***	−0.193	0.029	0.053	0.137 *	0.054
**Independent Variable**						
ESM Visibility (T1)	0.264 ***	0.036	0.015	0.049	0.023	0.050
**Mediator Variable**						
Workplace Impostor Thoughts (T2)			−0.288 ***	0.043	−0.245 ***	0.044

Notes: n = 447. Statistics reported are unstandardized regression coefficients. T1 denotes the variable was measured at Time 1; T2 denotes the variable was measured at Time 2. SE denotes Standard Errors. * *p* < 0.05 (two-tailed), ** *p* < 0.01 (two-tailed), *** *p* < 0.001 (two-tailed).

**Table 5:** Path analysis results for moderation effects and moderated mediation effects.

Variables	Workplace Impostor Thoughts (T2)		Knowledge Sharing (T2)		Workplace Well-Being (T2)	
	*b*	*SE*	*b*	*SE*	*b*	*SE*
**Control Variables**						
Gender	0.046	0.092	−0.122	0.089	−0.127	0.091
Age	0.067	0.075	−0.122	0.089	−0.127	0.091
Education	−0.031	0.086	−0.092	0.073	−0.014	0.074
Experience	−0.082	0.088	0.066	0.084	0.000	0.085
Organizational Tenure	−0.168 *	0.075	0.011	0.086	0.036	0.088
Position	0.074	0.054	0.243 **	0.073	0.103	0.075
ESM Use Hours	−0.112 **	0.038	0.029	0.053	0.137 *	0.054
**Independent Variable**						
ESM Visibility (T1)	0.259 ***	0.048	0.015	0.049	0.023	0.050
**Mediator Variable**						
Workplace Impostor Thoughts (T2)			−0.288 ***	0.043	−0.245 ***	0.044
**Moderator Variable**						
Social Comparison Orientation (T1)	−0.133 **	0.043				
**Interaction**						
ESM Visibility (T1) × Social Comparison Orientation (T1)	0.123 **	0.039				

Notes: n = 447. Statistics reported are unstandardized regression coefficients. T1 denotes the variable was measured at Time 1; T2 denotes the variable was measured at Time 2. SE denotes Standard Errors. * *p* < 0.05 (two-tailed), ** *p* < 0.01 (two-tailed), *** *p* < 0.001 (two-tailed).

**Table 6:** Mediation effects and moderated mediation effects based on 5000 bootstrapping replications.

Path	Indirect Effect	SE	95% CI
**Mediation effects**			
ESM→WIT→KS	−0.0520	0.012	[−0.0895, −0.0182]
ESM→WIT→WWB	−0.0429	0.0162	[−0.0773, −0.0419]
**Moderated mediation effects**			
ESM→WIT→KS			
High SCO (+1SD)	−0.106	0.027	[−0.159, −0.056]
Low SCO (−1SD)	−0.012	0.021	[−0.054, 0.029]
Differences (high − low)	−0.059	0.018	[−0.096, −0.027]
ESM→WIT→WWB			
High SCO (+1SD)	−0.087	0.024	[−0.137, −0.044]
Low SCO (−1SD)	−0.010	0.018	[−0.048, 0.022]
Differences (high − low)	−0.049	0.017	[−0.084, −0.019]

Abbreviations: ESM, ESM visibility; WIT, workplace impostor thought; SCO, social comparison orientation; KS, knowledge sharing; WWB, workplace well-being.

## Data Availability

Data are available from the corresponding author upon reasonable request.
